# Editorial: Improving the safety of cell therapy products by suicide gene transfer

**DOI:** 10.3389/fphar.2015.00174

**Published:** 2015-08-25

**Authors:** Elodie Bole-Richard, Marina Deschamps, Christophe Ferrand, Eric Robinet

**Affiliations:** ^1^Institut National de la Santé et de la Recherche Médicale UMR 1098, Etablissement Français du Sang Bourgogne/Franche-ComtéBesançon, France; ^2^SFR FRD 4234 Ingénierie et Biologie Cellulaire et Tissulaire, Université de Franche-ComtéBesançon, France; ^3^Institut National de la Santé et de la Recherche Médicale UMR 1110, Institut de Recherche sur les Maladies Virales et HépatiquesStrasbourg, France; ^4^Institut Hospitalo-Universitaire de StrasbourgStrasbourg, France

**Keywords:** suicide gene therapy, adoptive immunotherapy, adoptive cell therapy, inducible caspase 9, herpes simplex virus thymidine kinase

With the clinical demonstration of the efficacy of immune checkpoints inhibitors, immunotherapy has been considered in 2013 to be the major breakthrough for cancer therapy (Couzin-Frankel, 2013). Cell therapy and adoptive cell therapy (ACT), a method based on administration of immunocompetent cells in order to induce a therapeutic response in patients with solid cancer, hematologic malignancies, or immune diseases, have been widely and successfully tested in clinic for the past few years (Rosenberg and Restifo, 2015). Allogeneic hematopoietic stem cell transplantation (HSCT) is the most potent and consolidated ACT approach for the immunotherapy of hematologic malignancies (Tsirigotis et al., 2014). Alternative ACT approaches rely on the selection, expansion and possibly gene modification of autologous or allogeneic immune cells, such as regulatory or effector T cells, NK cells, or dendritic cells. Chimeric antigen receptor (CAR)-T cells transfer is a very promising therapy. CARs are antigen-binding domains linked to T-cell signaling domains, allowing for a specific recognition of cell surface antigens independently of the Major Histocompatibility Complex. Many clinical trials have demonstrated the efficiency of genetically modified T cells (GMCs) to treat cancers, including CAR-T cells. However, severe side effects of allogeneic HSCT or ACT, including the induction of graft-vs.-host disease (GvHD) or on target, off tumor effects limit the use of ACT, prompting researches to improve the safety of cell therapy products.

Suicide gene therapy was initially developed with the aim of introducing in cancer cells a gene whose activation by a prodrug would provide a toxic effect toward target cells and surrounding cancer cells, an approach beyond the scope of this Research Topic. The proof-of-concept of the transfer of a suicide gene (encoding the Herpes Simplex virus thymidine kinase, HSV-tk), not directly in cancer cells but into donor T cells, prior allogeneic HSCT, in order to control their alloreactivity was first described in 1994 (Tiberghien et al., 1994). Since then, clinical trials have demonstrated the feasibility of this approach. Several suicide systems have been developed to secure cell therapy and anti-tumor immunotherapy approaches in order to avoid adverse events. In this context, the aim of this Research Topic in Frontiers is to provide a state of the art of available tools to secure cell therapy products.

Jones et al. (2014) present different suicide gene families and summarize clinical results obtained to date. Suicide genes are classified according to the mechanism of action developed: mAb-mediated, such as CD20 antibody, metabolic with HSV-tk and dimerization-induced with inducible Caspase-9 (iCasp9). HSV-tk has been clinically validated in allogeneic HSCT in order to kill T cells in case of GvHD, by administration of its prodrug, ganciclovir (GCV). Briefly, GCV treatment resulted in significant reduction in the number of circulating GMCs with no acute toxicity. Some disadvantages appeared such as elimination of GMCs in several days, an immunogenicity of non-human transgene, a possible interference of GCV as antiviral in case of Cytomegalovirus (CMV) reactivation. So, alternative suicide genes have be proposed in order to decrease immunogenicity and to accelerate GMC elimination. Brenner and collaborators developed the iCasp9 suicide gene. Its prodrug, AP1903, a synthetic and non-toxic ligand, leads to iCasp9 dimerization and triggering of apoptotic pathway (Tey et al., 2007). This system allows rapid and specific GMCs' elimination and is currently tested in clinical trials. To date, the iCasp9 remain one of the most promising suicide gene secure system. Another interesting suicide gene is the human CD20, a B-cell membrane marker that can be used both as a selection marker, allowing for the immunomagnetic sorting of transduced cells and as a suicide gene, through the *in vivo* depletion of CD20-positive cells with CD20 monoclonal antibodies, such as Rituximab. Respective pros and cons of each approach are presented by Jones et al.

The first suicide gene historically clinically used for ACT was the HSV-tk gene (Bonini et al., 1998; Tiberghien et al., 2001). The group of Bordignon and Bonini, in Milano, has the greatest clinical experience of HSV-tk GMCs infusion. Their major contribution in the field of suicide gene therapy after allogeneic HSCT is presented by Greco et al. (2015). The Milano group developed Herpes Simplex Virus thymidine Kinase (HSV-tk) suicide gene, strategy, and application and demonstrated (i) the possibility to control T-cell alloreactivity after allogeneic HSCT by infusion of ganciclovir (GCV), (ii) the ability of GMCs to provide an anti-leukemic effect and (iii) their ability to improve post-transplant immune cell reconstitution, while preserving their protective functions toward viral and bacterial infections. Their clinical trials involved several Europeans teams, including the one of Weissinger et al. who, in their paper (Weissinger et al., 2015), present the long term follow up of patients after transfusion of HSV-TK transduced T-cells in the context of allogenic HSCT. Clinical trials have been done between 2002 and 2007. Patients who developed a GvHD have been successfully treated with GCV and they did not observe non-functional HSV-tk gene. Today, some patients are still alive but no HSV-tk expression was detected in these patients. These studies demonstrated safety, efficacy and feasibility of using HSV-tk GMCs. Although major contributions have been provided by clinical trials using HSV-tk-engineered T cells, a major issue remaining to be solved is related to the monitoring of GMCs in patients. In this article, Eissenberg et al. (2014) suggest to use HSV-tk for monitoring functions in order to localize GMCs. Using ^18^F-9-(4-fluoro-3-hydroxymethylbutyl) guanine (^18^FHBG), it is possible to follow and monitor GMCs with PET/CT scans in order to know if cells reach their target.

Given the limitations of the HSV-tk/GCV system, a second generation of suicide gene has recently emerged, whose leader is the iCaps9. As noticed by Gargett and Brown, CAR therapy show promising results due to partial or complete remissions observed upon infusion of CD19 CAR T-cells to patients with B-cell malignancies or after GD2 specific T-cell administration to patients with neuroblastoma (Gargett and Brown, 2014). Over 83 clinical trials using CAR T cells have been registered (*www.clinicaltrials.gov*). However, many serious adverse events such as cytokine storm and death can occur and have been reported. These problems are independent of the number of co-stimulatory domains and some of them are already known with chemotherapy and targeted therapies. However, today, it is necessary to improve the safety of cellular therapy products in general and particularly of CAR T cells. As presented by Gargett and Brown, the iCasp9 approach is a highly promising approach offered for securing CAR T cells. The association of CAR gene transfer with suicide gene transfer into T cells is currently reaching clinical application, but a similar approach on NK cells is still in preclinical development. To finish, Glienke and collaborators investigated CAR therapy in NK cells and the necessity to use suicide switches (Glienke et al., 2015). A lot of cancer antigens are targeted by CAR T cells in clinical trials. However, authors highlight the use of genetically modified NK cells with CAR, due to advantages of NK cells. Today, only two clinical studies are evaluating CAR-expressing NK cells. In this paper, they also noticed the importance to use suicide gene in order to secure NK-cell therapies.

As a conclusion, use of suicide system to secure new cell therapies, especially for those using gene-modified cells, is an important approach in order to improve cell therapy safety and decrease adverse effects. This Research Topic show that many improvements have been obtained during suicide gene development and that this technology has reached a maturity that should allow now considering more systematically its use when developing innovative cell therapies, including not only ACT, but also induced pluripotent stem cells- or mesenchymal stem cell based therapies.

## Conflict of interest statement

The authors declare that the research was conducted in the absence of any commercial or financial relationships that could be construed as a potential conflict of interest.
